# Use of an Online Community to Develop Patient-Reported Outcome Instruments: The Multiple Sclerosis Treatment Adherence Questionnaire (MS-TAQ)

**DOI:** 10.2196/jmir.1687

**Published:** 2011-01-24

**Authors:** Paul Wicks, Michael Massagli, Amit Kulkarni, Homa Dastani

**Affiliations:** ^2^Novartis Pharmaceuticals CorporationEast Hanover, NJUnited States; ^1^PatientsLikeMeResearch & DevelopmentCambridge, MAUnited States

**Keywords:** Medication adherence, multiple sclerosis, online communities

## Abstract

**Background:**

Patients with multiple sclerosis (MS) may face barriers, such as treatment fatigue, memory problems, or side effects, that may influence their adherence to medication.

**Objective:**

The objective of our study was to use an online community to develop a self-report questionnaire to quantify adherence and barriers to achieving adherence, that is specific to MS disease-modifying treatments (DMTs) and predictive of missed doses.

**Methods:**

A review of the scientific literature and analysis of discussions between MS patients on PatientsLikeMe.com were used to generate survey items salient to patients. Cognitive debriefing was used to refine the items. The Multiple Sclerosis Treatment Adherence Questionnaire (MS-TAQ) contains 30 questions in three subscales: Barriers, Side Effects, and Coping Strategies.

**Results:**

MS patients completed an online survey (response rate: 431 of 1209 invited, 35.7%). Between 16% (14/86) and 51% (51/100) of MS patients missed at least 1 dose of their DMT in the previous 28 days, with significant between-treatment differences. The MS-TAQ Barriers scale was positively correlated with the proportion of doses missed (*r* = .5), demonstrating a stronger relationship between adherence and perceived barriers than was found with clinical or demographic variables (*r* ≈ .3). The Coping Strategies subscale was negatively correlated with missed doses (*r* = -.3), suggesting that use of more coping strategies is associated with higher adherence.

**Conclusions:**

Online communities can provide domains of interest and psychometric data to more rapidly develop and prototype patient-reported outcome instruments. The MS-TAQ offers patients and clinicians a simple method for identifying barriers to adherence, which may then be targeted through interventions.

## Introduction

The World Health Organization estimates an average rate of only 50% adherence for patients with chronic medical conditions [1]. In diabetes the implications for nonadherence are clear; every 10% increase in medication adherence leads to a 0.1% decrease in glycosylated hemoglobin levels [2], and each 1% decrease in glycosylated hemoglobin leads to a 21% decrease in risk of death from diabetes [3]. The mechanisms and consequences of nonadherence to disease-modifying treatments (DMTs) in multiple sclerosis (MS) have attracted less attention, and it remains unclear what level of adherence is required to achieve maximum benefit [4]. Clinicians are without a means of accurately quantifying adherence to DMTs, regardless of the therapy chosen, and patients are without a means of sharing information with their physicians about barriers they experience to being fully adherent.

There is widespread agreement, based on clinical and magnetic resonance imaging assessments, that early DMT use in MS reduces the number of relapses and delays disease progression [5-7]. Following acceptance of therapy, at least two issues impede DMT use. First is DMT discontinuation; around 20% of patients discontinue their DMTs in the first year, usually after the first 6 months on treatment [8]. Reasons for discontinuation include perceived lack of effectiveness, lower levels of disability, injection-site reactions, and other side effects [9,10]. Second is nonadherence or missed doses – that is, taking a treatment less frequently than prescribed or failing to follow prescriber guidance. Adherence can be ascertained through a pill count, lab test, medical chart, self-report, collateral report, or electronic monitor [11]. A recent study comparing the performance of a medication event monitoring system (MEMS) to patient diaries and retrospective self-report found an acceptable correlation between the two (*r* = .7), though self-report was noted to systematically underreport the proportion of nonadherent patients [12]. The Global Adherence Project (GAP), a large international observational study (N = 2648) that employed retrospective self-report, recently reported that 75% of patients were perfectly adherent to their DMT over 4 weeks [13]. They found that patients who had been on therapy longer, were male, and had longer disease duration were more likely to have skipped at least 1 dose.

Reasons for nonadherence in MS are complex; in an online survey of nearly 800 MS patients [14], the most important factors identified by patients were forgetting to administer the DMT (58%), not feeling like taking the DMT (22%), or feeling tired of taking the DMT (16%). Other factors included skin reactions (5%), pain at injection sites (7%), injection-related anxiety (3%), and needing someone else to administer the injection (4%). Similarly, the GAP study found that 32% of patients provided needle-based barriers as reasons for missing a dose, but that forgetting was still the strongest factor (identified by 50% of nonadherent patients).

Emotional and cognitive issues may also be important; patients with decreased memory function, or increased levels of anxiety or fatigue have lower levels of adherence; patients with a current mood or anxiety disorder were almost 5 times more likely to be nonadherent [15]. In a prospective study, Tremlett et al found a higher number of missed doses among patients with more frequent DMT injections and heavier alcohol use, but found no relationship with side effects resulting from treatment [16]. Using a health beliefs model to understand adherence, Turner et al found that perceived DMT effectiveness, but not barriers to adherence, predicted adherence [4].

In terms of consequences of nonadherence in MS, patients who discontinue treatment are more likely to experience progression of their disability [8]. Furthermore, claims data suggest that gaps in medication availability are associated with a 1.5 to 2 times odds ratio of subsequent admission to hospital [17,18]. The GAP study found that adherent patients had a higher quality of life and lower neuropsychological impairment, although the direction of causality is unclear [13].

Despite relatively consistent barriers to adherence in the literature, scale development in this area has historically focused on injection pain and perceived needle sharpness due to needles or infusions as the means of DMT delivery [19-21]. More patient-centered approaches have been educational in nature or recommended psychosocial interventions, but have been without structured assessment tools [22-24]. A recent editorial pointed out that the “core issue of adherence” is identifying the reasons why patients have decided to be nonadherent, and that much of the literature fails to illuminate the spectrum of behavior between perfect compliance and nonadherence [25].

Online communities may present an opportunity to illuminate unmet patient needs that are outside those identified in the scientific literature or in clinic visits. We sought to build an MS-specific understanding of adherence to DMTs by developing a scale from patients’ own descriptions of their barriers to adherence, called the Multiple Sclerosis Treatment Adherence Questionnaire (MS-TAQ). We hypothesized that perceived effectiveness and barriers to adherence would predict self-reported adherence. We also hypothesized that patients using coping strategies to minimize side effects would have better self-reported adherence.

## Methods

### Scale Development

PW reviewed the scientific literature in June 2009 in EMBASE and Medline using major and minor headings for the terms multiple sclerosis, compliance, patient compliance, adherence, treatment refusal, non-adherence, and nonadherence. Reference lists were reviewed for additional sources. We identified the following relevant themes as important to adherence: discontinuation, forgetting to take medication, perceived lack of effectiveness, pain, needle phobia/anxiety, adverse reactions, support and patient education, availability of help with injecting, and stigma or reminders of disease.

To further identify relevant themes regarding adherence from the patients’ own discussions, MM and PW conducted a computer-assisted search of the PatientsLikeMe MS community online message board (forum), which as of June 30, 2009 contained 373,345 posts across 23,224 threads, contributed by 4844 unique patients. Patients generate and discuss a range of unprompted topics relevant to managing their condition. PW and MM generated a list of relevant search terms (eg, inject, shot, site reaction, and pain) and applied them to a random sample of 1000 posts; these were reviewed for additional terms and applied to another sample of 4000 post, which were also reviewed for additional terms and refined to eliminate terms that were not discriminating discussions relevant to adherence. For example, the term pain usually referred to symptoms of MS rather than injection-related pain, but sting or soreness normally referred to adherence issues. A final list of 49 terms was reapplied to the original samples (5000 posts) plus additional random samples for a total of 80,000 forum posts. Of these, 6.27% (5019/80,000 posts) contained at least a single mention of one term, but manual review by MM showed that many of these were not relevant (eg, discussions of how to apply for disability insurance rather than discussions of the nature of disability). However, posts with two or more terms in them were almost always relevant to adherence, so we focused on the 1.57% of posts (1254/80,000) that this applied to. The posts containing the most search terms tended to come from long-term patients explaining their own experience to patients with a more recent diagnosis and offering their own advice.

The most obvious themes to arise overlapped those from the scientific literature. For example, a number of studies have found a link between adherence and perceived effectiveness [4,14,15,26-28], and this was a theme readily apparent in patients’ forum discussions; for example, one patient wrote “*You never know ‘for sure’ if they are helping prevent future flares...but I s’pose it[’]s not a leap of faith that if you have no flares in the future, it[’]s due to the DMTs.”*

However, our qualitative analysis of online community data generated three additional issues not previously described as drivers of adherence in the literature. First, we identified a range of coping strategies being used by patients to modulate consequences of their DMT – for example, “*I take ibuprofen with my injection and sleep through any side effects there may be.”* Second, patients’ interpretations of the current severity and impact of their barriers or side effects can be strongly influenced by their previous exposure to other DMTs, which provides a contextual anchoring effect to their current problems. For example, a patient might say “I’m experiencing some side effects on treatment X but they’re much less of a problem than what I experienced with treatment Y.” Third, we found that patients’ experience of some side effects, such as injection-site reactions, waxed and waned over time. While cross-sectional studies might consider them to be present or absent, it was clear that some problems arose some time after treatment commencement while others resolved spontaneously.

On the basis of themes identified in this process, an experienced survey designer drafted question stems and responses. Items were grouped into three subscales, each with a different response format: DMT-Barriers quantifies the extent to which the patient rated 13 barriers to adherence as important reasons for nonadherence (asked only of patients who missed at least 1 dose in the previous 28 days, 4-point scale from “not important at all” to “extremely important” in missing or forgetting a dose); DMT-Side Effects describes the frequency of 10 side effects (asked of all patients, 5-point scale from “never” to “all or nearly all of the time”); and DMT-Coping Strategies is a count of 7 coping mechanisms used by the patient to reduce side effects (eg, using an ice cube on the injection site, asked of all patients, binary yes/no response for “in the past 4 weeks (28 days) did you usually...”).

Five female white patients participated in real-world cognitive debriefings after completing a draft version of the questionnaire, according to recommended guidelines [29]. Patients reported that questions were clear and simple, but suggested changing the reference period from “the past 30 days” to “the last 4 weeks (28 days)”. 

### Participants

Patients reporting a diagnosis of MS were recruited from an online community, PatientsLikeMe. The site has been described previously [30-32]. This online system allows patients with serious illnesses to share their symptoms, treatments, and outcome measures of interest (functional disability, weight, quality of life) in an open medical platform. Patients evaluate their perceptions of treatments, including perceived effectiveness, side effects, burden, and adherence, and can also participate in clinical research. The website features a survey tool, PatientsLikeMeLens, which allows selection of participant lists and online administration of surveys.

The following information is provided to comply with the Checklist for Reporting Results of Internet E-Surveys [33]. Patients who had logged on to the site in the preceding 90 days were randomly selected to participate, from an overall pool of approximately 15,000 registered MS patients. On December 21, 2009, six blocks of approximately 200 survey invitations were sent to patients reporting current DMT use on their patient profiles for the following groups: not currently taking a DMT (No DMT), glatiramer acetate (GA; Copaxone, Teva Pharmaceutical Industries Ltd, Petach Tikva, Israel), interferon beta-1a intramuscular injection (IFB-1a IM; Avonex, Biogen Idec, Weston, MA, USA), interferon beta-1a subcutaneous injection (IFB-1a SC; Rebif, EMD Serono Inc, Rockland, MA, USA), interferon beta-1b subcutaneous injection (IFB-1b SC, Betaseron, Bayer Healthcare, Leverkusen, Germany), and natalizumab infusion (Tysabri, Biogen Idec). The invitation was sent as a private message within the PatientsLikeMe community, with a customized research invitation message arriving in members’ email inboxes.

New private messages trigger an automated email to patients’ email accounts (unless they have opted out of being contacted in this way). Sampled patients had their own password-protected login; they could complete the survey only once, and we have tools to prevent multiple accounts originating from the same location, including account registration, cookies, and internet provider tracing. Therefore, we have more confidence in our denominators than might be found using an “open” survey method. The survey was voluntary to complete and was not mandatory to complete in order to continue using the other features of the site. No incentives were offered; question order was not randomized; certain items only appeared conditional on previous responses (ie, were “adaptive”) to minimize respondent burden (see Multimedia Appendix 1); and the total number of questions and screens varied by participants’ own responses.

Following initial contact, a reminder message was sent within a week to those who had not yet completed the survey; patients who had only partially completed the survey could reaccess it through the original private message (or reminder message) to complete their survey. Once opened, the survey had a “back” button that allowed participants to change their earlier answers. Only data from completed questionnaires are presented here. The study was approved by Western Institution Review Board (WIRB), Olympia, WA, USA (Study 1111772). Patients gave informed consent electronically.

Members of PatientsLikeMe join the site with the expectation that they will be participating in research. The recruitment message outlined the purpose of the study and reminded patients that they were under no obligation to participate, that their aggregated results may be published, and that the survey should take about 20 minutes to complete. It was sent from the user account for PW, who can easily be contacted by potential participants from within the PatientsLikeMe system.

User data were protected in accordance with PatientsLikeMe’s internal security standard operating procedures, which include password protection, deidentification of locally held data files, regular automated backup, and physical protection of information technology hardware.

### Adherence

Patients were asked on how many of the previous 28 days they were supposed to take a dose, whether they missed or forgot any doses, and, if so, how many. For those who missed at least 1 dose, missed dose ratio (MDR) is reported as the number of doses missed divided by the number of prescribed doses over a 28-day period. For example, a patient missing 1 shot of daily GA in 28 days would have an MDR of 0.04, while a patient missing 1 shot of weekly IFB-1a IM would have an MDR of 0.25. For between-treatment group comparisons, MDR is provided for all patients; those who did not report missing a dose were coded as having an MDR of 0.

### Survey Items

A complete copy of the questions presented to participants is included in Multimedia Appendix 1. In addition to the MS-TAQ, the survey included demographic information (age, weight, height, sex), MS symptoms (memory, concentration/attention, comprehension, expression, anxiety, depression, vision problems), burden of illness items (ability to work for pay, ability to meet household responsibilities), current DMT, DMT history, perception of side effects, DMT duration, MDR, ability to grasp an injector, method of injection, need for assistance with injection by others, use of manufacturer’s support service, expectations and perceptions of DMT effectiveness, and overall satisfaction with DMT.

We included a self-report measure of functional impairment, which has been in use on the PatientsLikeMe site since its launch in 2007. The MS Rating Scale asks patients to rate their current level of disability in seven domains: walking, arm function, vision, speech, swallowing, cognition, and sensation. Response options are “No symptoms or disability in this specific area (0),” “None - Aware of symptoms but no functional disability (1),” “Mild - Mild disability but not requiring help from others (2),” “Moderate - Moderate disability that requires some help from others (3),” and “Total - Total disability and help always required (4)”. Summing the responses produces a scale with a range of 0-28, which is normed to 0-100 (higher score represents greater disability).

### Statistical Analysis

Statistical analysis was performed using (SPSS) version 17.0 (IBM Corporation, Somers, NY, USA). Group comparisons were assessed using one‐way analysis of variance where normally distributed, or Kruskal‐Wallis tests where non‐parametric. All correlations shown are nonparametric Spearman correlations due to the ordinal nature of the scales. Alpha for significance was set at *P* = .05 (two‐tailed). A logistic regression model was used to estimate the net effect of patient factors and behaviors on the odds of missing at least 1 dose. A linear regression model was used to estimate the net effect of factors influencing the MDR. In both cases we used generalized estimating equations, and the Wald chi-square to test model effects.

## Results

### Participants

In December 2009, survey invitations were sent to 1209 members of the MS community in six blocks of about 200 patients stratified by DMT usage; 41.9% patients responded (507/1209) and complete responses were analyzed for 35.7% (431/1209). We excluded the following from further analysis: 62 patients who did not answer all questions, 3 who were taking mitoxantrone, which was too small a group to analyze, and 11 who provided inconsistent data about their DMT use. Demographics are provided in Table 1. The majority of respondents (311/431, 72.2%) reported a relapsing-remitting form of MS, 10% (45/431) reported that their MS was secondary progressive, 10% (44/431) did not know their MS type, 4% (17/431) reported primary progressive MS, and 4% (16/431) reported progressive relapsing MS. The primary analyses were repeated separately for the relapsing-remitting group and did not materially change the main results of the study; data presented here represent all patient-reported MS subtypes.

There were no significant differences in response rate by sex (c^2^_3_ = 4.5, *P* = .02). There were significant differences for age (F_3,1205_ = 4.860, *P* = .002) between responders and nonresponders in the community. Post hoc tests showed that patients who completed the survey were older (mean difference 2.3 years, 95% CI 0.5-4 years, *P* = .004) than those who did not respond.

**Table 1:** Respondent demographics by disease-modifying treatment (DMT)

	GA (n = 101)^a^	IFB-1a IM (n = 87)^b^	IFB-1a SC (n = 81)^c^	IFB-1b SC (n = 63)^d^	Nat (n = 58)^e^	No DMT (n = 41)	Between-DMT significance	Total average (N = 431)
Mean (SD) age, years	47 (11)	48 (11)	44 (10)	47 (9)	44 (10)	48 (11)	F_5,425_ = 2.230, *P* = .05	46 (10)
Sex, % female	85 (84%)	68 (78%)	61 (75%)	57 (91%)	41 (71%)	30 (73%)	c^2^_5_ = 10.7, *P* = .06	431 (79%)
Mean (SD) BMI^f^, kg/m^2^	29 (7)	27 (6)	30 (7)	29 (6)	27 (6)	27 (6)	F_5,415_ = 3.314, *P* = .01	28 (7)
First DMT? n (%)	73 (72%)	73 (84%)	50 (62%)	47 (75%)	3(5%)	NA^g^	c^2^_4_ = 107.0, *P* < .001	246 (57%)
Median DMT duration, months	25	22	22	25	16	NA	c^2^_4_ = 19.1, *P* < .001	22
Mean (SD) time since onset, years	10 (9)	11 (10)	7 (8)	11 (10)	15 (9)	14 (8)	F_5,418_ = 5.295, *P* < .001	11 (9)
Mean (SD) time since diagnosis, years	6 (7)	7 (7)	5 (6)	8 (8)	11 (7)	10 (7)	F_5,415_ = 5.870, *P* < .001	7 (7)

^a^ GA: glatiramer acetate.

^b^ IFB-1a IM: interferon beta-1a intramuscular injection.

^c^ IFB-1a SC: interferon beta-1a subcutaneous injection.

^d^ IFB-1b SC: interferon beta-1b subcutaneous injection.

^e^ Nat: natalizumab infusion.

^f^ BMI: body mass index.

^g^ NA: not applicable.

To assess biases in our sample we compared the demographics of our sample (Table 1) to the Sonya Slifka MS study by Minden et al [34]. Our populations appeared similar for sex (PatientsLikeMe: 79.3% (342/431) vs Minden et al: 77%) but ours were slightly younger (PatientsLikeMe mean age 47 years, SD 10 vs Minden et al: 51 years, SD 11), and had been symptomatic for less time (PatientsLikeMe mean duration since onset : 11 years, SD 9 vs Minden et al: 18 years, SD 11). Relative to Minden et al, our sample had a higher proportion of patients with relapsing-remitting MS (72.2% (311/431) vs 58%), a lower proportion with secondary progressive MS (10% (43/431) vs 25%), and lower proportion with primary progressive MS (4% (17/431) vs 13%), but similar proportions with progressive relapsing MS (4% (16/431) vs 5%). However, given that we were selecting patients who were using DMTs, the relatively high proportion of patients with relapsing-remitting MS is unsurprising.

### Adherence

**Figure 1:**
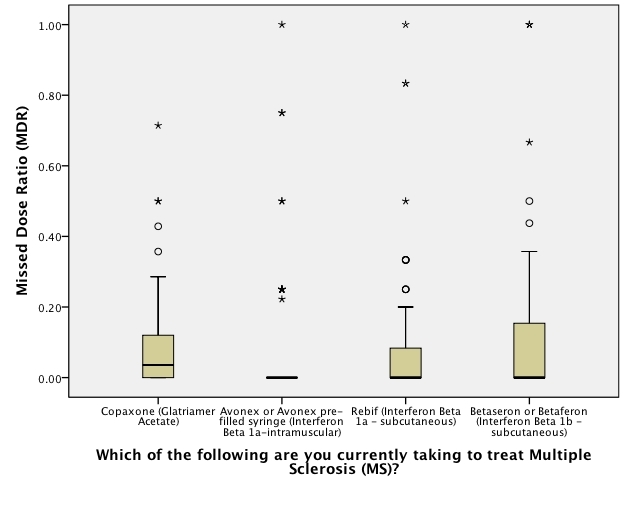
Significant differences in missed dose ratio for all patients in the past 28 days; 0.00 = fully adherent, 1.00 = missed every prescribed dose(circles: outliers >1.5 but <3 interquartile ranges [IQRs]; asterisk: >3 IQRs from nearest edge of boxplot; bolded symbols: >1 point in same place)

**Figure 2:**
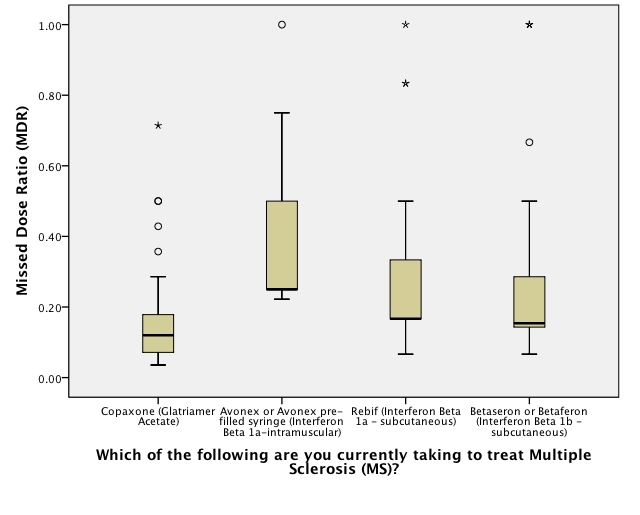
Significant differences in missed dose ratio for patients who reported missing a dose in the past 28 days; 0.00 = fully adherent, 1.00 = missed every prescribed dose (circles: outliers >1.5 but <3 interquartile ranges [IQRs]; asterisk: >3 IQRs from nearest edge of boxplot; bolded symbols: >1 point in same place)

There were significant differences between the proportions of patients missing a dose in each treatment group (see Table 2). Overall, between 16% and 51% of MS patients missed at least 1 dose of their DMT. Seven patients missed a dose of natalizumab, and open-text responses showed that their physician had changed their dosing schedule to every 6 or 8 weeks in response to safety concerns. Therefore, the 28-day time frame of the original questions is rendered invalid; data on the natalizumab patients was therefore excluded from further analysis related to adherence. Figure 1 shows the distribution of MDR between DMTs across all patients. Figure 2 shows the distribution of MDR between DMTs across only those patients who missed at least 1 dose.

A logistic regression model was constructed to study the net impact of personal and disease factors on the likelihood of having missed a dose of their DMT in the preceding 28 days. Patients were more likely to have missed at least 1 dose in the past 28 days if they had a disease type other than relapsing-remitting (*P* = .002), lower levels of disability (*P* = .03), or a history of taking more than one DMT in the past (*P* = .04). There were no significant associations with age, sex, body mass index, disease duration, time on treatment, or difficulty grasping the injector.

**Table 2:** Number of missed doses in the preceding 28 days by disease-modifying treatment (DMT)

	GA (n = 100)^a^	IFB-1a IM (n = 86)^b^	IFB-1a SC (n = 81)^c^	IFB-1b SC (n = 63)^d^	Nat (n = 58)^e^	Between-DMT significance
Typical dosing	Daily	Weekly	Every 3 days	Alternate days	Monthly	NA^f^
Prescribed doses, median (range)	28 (0-28)	4 (0-9)	12 (3-16)	14 (7-21)	1 (1-1)	NA
Patients who missed a dose, n (%)	51 (51%)	14 (16%)	25 (31%)	31 (49%)	7, NA^g^	c^2^_4_ = 63.0, *P≤* .001
Missed doses, median (range)	3 (1-20)	1 (1-3)	2 (1-10)	2 (1-14)	NA	NA
Nonadherent MDR ^h^, mean	0.16	0.41	0.29	0.28	NA	c^2^_3_ = 24.2, *P≤*.001
Nonadherent MDR ^h^, median (range)	0.12 (0.04-0.71)	0.25 (0.22-1.0)	0.17 (0.07-1.0)	0.15 (0.07-1.0)	NA	
All patients’ MDR, mean	0.08	0.07	0.09	0.14	NA	c^2^_3_ = 19.4, *P≤*.001
All patients’ MDR, median (range)	0.04 (0.00-0.71)	0.00 (0.00-1.00)	0.00 (0.00-1.00)	0.00 (0.00-1.00)	NA	
Managed to inject 100% of each dose taken?	85 (85%)	83 (97%)	78 (96%)	55 (87%)	54 (93%)	c^2^_4_ = 12.2, *P* = .02

^a^ GA: glatiramer acetate.

^b^ IFB-1a IM: interferon beta-1a intramuscular injection.

^c^ IFB-1a SC: interferon beta-1a subcutaneous injection.

^d^ IFB-1b SC: interferon beta-1b subcutaneous injection.

^e^ Nat: natalizumab infusion.

^f^ NA: not applicable.

^g^ Excluded from further analysis due to altered dosing range of 6-8 weeks per transfusion, rendering the 28-day window inapplicable.

^h^ MDR: missed dose ratio.

### Psychometric Performance of MS-TAQ

Psychometric performance of the MS-TAQ subscales is shown in Table 3. Cronbach alpha was acceptable for DMT-Barriers and DMT-Side Effects but was low for DMT-Coping Strategies; this may have been due to the limited range of the scale and the binary response options.

**Table 3:** Multiple Sclerosis Treatment Adherence Questionnaire (MS-TAQ) subscale characteristics

MS-TAQ subscale	Number of items	Score		Cronbach alpha	Between-DMT^a^ significance
		Mean (SD)	Range		
DMT-Barriers	13	9 (7)	0-39	.82	F_3,117_ = 1.236, *P* = .300
DMT-Side Effects	10	12 (9)	0-40	.86	F_4,394_ = 24.498, *P* < .001
DMT-Coping Strategies	7	1 (1)	0-7	.40	c^2^_24_ = 101.4, *P* < .001

^a^ DMT: disease-modifying treatment.

### Predictors of Missed Dose Ratio in Nonadherent Patients

Among nonadherent patients, a higher MDR was associated with lower DMT convenience (*r* = .33, *P* < .001), lower treatment satisfaction (*r* = -.30, *P* = .001), higher levels of anxiety (*r* = .21, *P* = .02), and higher levels of depression (*r* = .21, *P* = .02). Notably, MDR was not correlated with disease severity as measured by the MS Rating Scale (*r* = -.01, *P* = .90), total symptom severity (*r* = .07, *P* = .50), time since diagnosis (*r* = -.004, *P* = .97), or time since symptom onset (*r* = -.08, *P* = .37). We found no significant correlation between MDR and expectations of effectiveness (*r* = -.04, *P* = .66) or current perceived effectiveness (*r* = .17, *P* = .07), nor with the discrepancy between the two (*r* = -.17, *P* = .06).

However, there was a stronger correlation between MDR and the DMT-Barriers subscale of the MS-TAQ (*r* = .50, *P* < .001). The DMT- Coping Strategies subscale also correlated (negatively) with MDR (*r* = -.30, *P* = .003), suggesting that using a higher number of coping strategies was associated with better compliance. DMT-Side Effects was not significantly correlated with MDR (*r* = .10, *P* = .26).

A linear regression model was used to estimate the net effects of personal and behavioral factors on the MDR. Each point scored on the DMT-Barriers subscale of the MS-TAQ was associated with a 1% *increase* in MDR. Each point change on the DMT-Coping Strategies scale was associated with a 4% *decrease* in MDR.

## Discussion

Using qualitative and quantitative data sourced from an online community, we developed the MS-TAQ, a rating scale that quantifies the barriers to adherence, side effects, and coping strategies experienced by MS patients. The Barriers subscale is a more powerful predictor of missed doses (*r* = .5) than an overall satisfaction question (*r* = .3). We confirmed that patients who used more coping strategies to ameliorate side effects were able to take more doses of their medication than those who did not, even if they were still not perfectly adherent.

As in other studies, we identified associations between nonadherence and DMT satisfaction [4,13,14], lower levels of disability [13,35], more barriers to adherence [14], previous history of DMT use [14], and anxiety and depression [14,15]. Like others, we did not find a strong association between adherence and demographic variables [4,12,14,16]. Although side effects in MS DMTs may be associated with discontinuation[8], there does not appear to be an obvious relationship with nonadherence [16]. Side effects are also a common reason for treatment discontinuation in other chronic conditions [36]. The lack of clear relationship in MS may be due to several reasons: first, all DMT options have side effects and it may be a case of learning to live with them; second, we identified that the coping strategies patients develop to live with these side effects were an important predictor of nonadherence; and third, DMTs are widely known to be effective in reducing disease activity and the symptoms of MS are too severe to ignore.

In our study, we observed no association between adherence and self-reported cognitive issues, though this may be because patient self-report may be relatively insensitive to memory problems and we did not use a neuropsychological test battery [15]. Our findings concur with those of Tremlett et al [16], confirmed by the GAP study [13], who found that DMTs with less frequent dosing regimens had better adherence. Tremlett et al found a higher proportion of patients who missed at least 1 dose (73%) than in our study (121/388, 31.2%), but the former was a prospective study with reporting of a 6-month period, whereas ours was 28 days. Their findings suggested an MDR of approximately 0.14 for IFB-1b (vs 0.14 in the current study), 0.13 (vs 0.09) for IFB-1a SC, and 0.06 (vs 0.08) for GA. The GAP study identified a slightly lower proportion of patients missing at least 1 dose in the preceding 4 weeks (25%) [13]. This may reflect methodological differences or, speculatively, could be related to the means of data capture; patients may be more willing to admit to nonadherence online.

Treadaway et al [14] found that, overall, their most frequently endorsed barrier was “forgetting,” but that in the least adherent patients this was lower, with “not feeling like taking injection” and “injection anxiety” being more significant problems. In our nonadherent patients, we also found that the main barriers were “Did not feel like taking my DMT” and “Tired of taking my DMT,” followed by “Memory problems.” This suggests that adherence aids that only provide reminders may still fail to tackle the barriers of patients with the poorest levels of adherence. More work needs to be done to understand how to help patients overcome “treatment fatigue” [35].

Over the preceding 28 days we found that 16%-51% of patients had missed at least 1 dose; there is evidence from the literature to believe the problem might be worse than that. For instance, Tremlett et al’s data suggest that nonadherence in our sample may continue to be a problem; they found that missed doses predict future nonadherence over a 6-month time frame [16]. Furthermore, through their comparison of self-report against passively collected MEMS data, Bruce et al suggested doubling self-report estimates of adherence to arrive at a more accurate estimate [12]. Interestingly, Bruce et al found that some MS patients seemed less likely to be adherent on Fridays and weekends, relative to weekdays [12]. Future research should address the pharmacokinetic consequences of different patterns of nonadherence – for example, skipping a dose every Friday versus not taking 4 doses for consecutive days in a month.

The imminent arrival of oral therapies [37-39] will require further attention to be devoted to measuring adherence in MS patients. The absence of injections and the potential for simplified dosing regimens such as a daily pill should be a significant improvement, but this assumption should be tested. Future research could adapt the MS-TAQ to measure barriers to adherence in oral medications.

As a self-report study conducted in an online population, this study is open to methodological limitations, such as selection bias, response bias, and the difficulty in knowing whether patients self-identifying as patients really do have MS. Similar approaches have been taken by online registries such as NARCOMS (North American Research Committee on Multiple Sclerosis) to answer questions of clinical relevance and real-world validation. Studies have found MS patients’ self-report data to have a high level of validity [40-42]. Our response rate was typical for an online study, and we have described how it differs from both nonresponders and the Sonya Slifka longitudinal study [34], but it is possible that the sample may have been biased in receiving more responses from those with barriers to adherence, which may limit the degree to which the findings can be generalized. We have also been reassured by the relative parity between our findings and those of the larger GAP study [13], which was ongoing at the same time as our own data collection. The use of an MDR with a reference of 28 days proved to be a limitation in measuring adherence to natalizumab transfusions, which may sometimes be spaced at 2-month intervals. The measure may also appear to inflate MDR for treatments with a weekly dosing schedule (as missing a single dose automatically means an MDR of 0.25). The implications for this are unclear, as it is quite uncertain what the exact consequences are for different levels of adherence for each DMT.

A number of studies have identified risk factors for missing a dose of a given medication. A novel aspect of the current study was that we found correlations between patient-generated barriers to adherence and the magnitude of their nonadherence. We also attempted to generate a more positive sense of adherence by identifying supportive coping strategies that may help patients overcome these barriers. It is hoped that the MS-TAQ will be a useful measure of adherence to be applied in MS in future studies, particularly if it can be validated against MEMS. Multimedia Appendix 2 includes a printable version of the MS-TAQ.

Generated by patients’ own experiences with adherence, the MS-TAQ can be used by health care professionals as a discussion aid, perhaps administered in the waiting room, in order to identify and overcome barriers to adherence. There is also potential to use online administration and feedback to empower patients to take responsibility for improving their own outcomes through improved adherence. Online communities have the potential to permit rapid development and psychometric validation of patient-reported outcomes.

## Multimedia Appendix 1

Copy of the MS-TAQ survey items presented to participants

## Multimedia Appendix 2

Printable version of the MS-TAQ for clinical use

## Abbreviations

DMT: disease-modifying treatment
GA: glatiramer acetate
GAP: Global Adherence Project
IFB-1a IM: interferon beta-1a intramuscular injection
IFB-1a SC: interferon beta-1a subcutaneous injection
IFB-1b SC: interferon beta-1b subcutaneous injection
MDR: missed dose ratio
MEMS: medication event monitoring system
MS: multiple sclerosis
MS-TAQ: Multiple Sclerosis Treatment Adherence Questionnaire
