# Supplementary material for: Use of an Online Community to Develop Patient-Reported Outcome Instruments: The Multiple Sclerosis Treatment Adherence Questionnaire (MS-TAQ)
Source: J Med Internet Res. 2011 Jan 24;13(1):e12. doi: 10.2196/jmir.1687 (PMC3221333; doi:10.2196/jmir.1687)
Supplement: Supplementary file 1 [file jmir_v13i1e12_app1.pdf]

PatientsLikeMe Survey Outline
Measuring Needle Fatigue in Multiple Sclerosis

You are being asked to participate in a research study about your experience and perception of the use of inject-able medications to treat Multiple Sclerosis and of the personal impact of this method of taking medication.

The purpose of this study is to better understand how needle-based treatments affect the user's quality of life and compliance with treatment recommendations. You will be asked to complete a 20-minute survey that includes questions about your experience and the impact of use of inject-able drugs to treat MS on your physical activities, social activities and emotional health. If you do not use injectable treatments, you will be asked about your perceptions or problems that you may have had in the past. The results from the study will be shared with the PatientsLikeMe MS community and may be presented in educational settings, at professional conferences, or published in professional journals in the field of medicine. No personal information will be presented in any way and all materials will be kept confidential.

No risks or discomforts are anticipated from taking part in this study. You can withdraw from the survey at any time without having your answers recorded. If you decide not to participate in the survey or if you decide to not complete the survey after you start it, you will still remain a member in good-standing of the PatientsLikeMe community.

If you have questions or concerns about this study, please contact PatientsLikeMe at research@patientslikeme.com .

If you have questions about your rights as a research subject or if you have questions, concerns or complaints about the research, you may contact:

Western Institutional Review Board (WIRB)

3535 Seventh Avenue, SW

Olympia, Washington 98502

Telephone: 1-800-562-4789 or 360-252-2500

E-mail: Help@wirb.com

WIRB is a group of people who perform independent review of research.

WIRB will not be able to answer some study-specific questions, such as questions about appointment times. However, you may contact WIRB if the research staff cannot be reached or if you wish to talk to someone other than the research staff.

Section 1. **About you**

1. **What is your age?**
   Key: *none*
   Format: Free-form text

   Please enter age in years.

2. **Are you:**
   Key: *none*
   Format: Multiple-choice
   - Female (Value: 1)
   - Male (Value: 2)

3. **What is your weight?**
   Key: *none*
   Format: Free-form text

   Please enter weight, in pounds without shoes

4. **What is your height?**
   Key: *none*
   Format: Free-form text

   Please enter your height in feet and inches, without shoes.

Section 2. **During the past 4 weeks (28 days) have you had any problems with the following?**
(Please check one answer for each)

1. **Your memory or remembering things**
   Key: *none*
   Format: Multiple-choice
   - None (Value: 1)
   - Mild (Value: 2)
   - Moderate (Value: 3)
   - Severe (Value: 4)

2. **Your ability to concentrate or pay attention to things**
   Key: *none*
   Format: Multiple-choice
   - None (Value: 1)
   - Mild (Value: 2)
   - Moderate (Value: 3)
   - Severe (Value: 4)

3. **Your ability to comprehend or understand things**
   Key: *none*
   Format: Multiple-choice
   - None (Value: 1)
   - Mild (Value: 2)
   - Moderate (Value: 3)
   - Severe (Value: 4)

4. **Your ability to think, express yourself, or figure things out**
   Key: *none*
   Format: Multiple-choice
   - None (Value: 1)
   - Mild (Value: 2)
   - Moderate (Value: 3)
   - Severe (Value: 4)

5. **Anxiety**
   Key: *none*
   Format: Multiple-choice
   - None (Value: 1)
   - Mild (Value: 2)
   - Moderate (Value: 3)
   - Severe (Value: 4)

6. **Depression**
   Key: *none*
   Format: Multiple-choice
   - None (Value: 1)
   - Mild (Value: 2)
   - Moderate (Value: 3)
   - Severe (Value: 4)

7. **Vision problems**
   Key: *none*
   Format: Multiple-choice
   - None (Value: 1)
   - Mild (Value: 2)
   - Moderate (Value: 3)
   - Severe (Value: 4)

Section 3. **Begin work outside of home questions**

1. **During the past 4 weeks (28 days), did you do any work for pay?**
   Key: WORK_PAY
   Format: Multiple-choice

   (Please check one answer)

- Yes (Value: 1)
- No (Value: 2)

Section 4. **Reason no work outside home**

Show if: (#WORK_PAY IS '2')

1. **Which of the following was the main reason you did not work for pay in the past 4 weeks (28 days)?**
   Key: *none*
   Format: Multiple-choice

   (Please check one answer)

   - Unable to work because of my Multiple Sclerosis (Value: 1)
   - Did not need to work (Value: 2)
   - Wanted to work, but do not have a job (Value: 3)

Section 5. **Days worked (q8)**

Show if: (#WORK_PAY IS '1')

1. **During the past 4 weeks (28 days), how many days did you do any work for pay?**
   Key: *none*
   Format: Free-form text

   Please enter the number of days here:

2. **Did you limit the number of days you worked because of your Multiple Sclerosis?**
   Key: LIMIT_DAYS_WORK_PAY
   Format: Multiple-choice
   - Yes (Value: 1)
   - No (Value: 2)

Section 6. **q8a followup**

Show if: (#LIMIT_DAYS_WORK_PAY IS '1') AND (#WORK_PAY IS '1')

1. **How many additional days would you have worked?**
   Key: *none*
   Format: Free-form text

   Please enter the number of additional days here:

Section 7. **q8b**

Show if: (#LIMIT_DAYS_WORK_PAY IS-ANSWERED)

1. **Did you limit the number of hours you worked on any day because of your Multiple Sclerosis?**
   Key: LIMIT_NUMBER_HOURS_WORK_FOR_PAY
   Format: Multiple-choice
   - Yes (Value: 1)
   - No (Value: 2)

Section 8. **q8c**

Show if: (#LIMIT_NUMBER_HOURS_WORK_FOR_PAY IS '1')

1. **About what percentage of the hours you were expected to work were you able to work during the past 4 weeks (28 days)?**
   Key: *none*
   Format: Free-form text

   Please enter the percent of expected hours you worked

Section 9. **q8d**

Show if: (#LIMIT_NUMBER_HOURS_WORK_FOR_PAY IS-ANSWERED)

1. **On the days you worked, about how many hours per day did you work?**
   Key: *none*
   Format: Free-form text

   Please enter hours per day you worked:

Section 10. **q8e**
On the days you worked...

Show if: (#WORK_PAY IS '1')

1. **were you able to work without making mistakes?**
   Key: *none*
   Format: Multiple-choice
   - Always (Value: 4)
   - Usually (Value: 3)
   - Sometimes (Value: 2)
   - Never (Value: 1)

2. **were you able to complete your work on time?**
   Key: *none*
   Format: Multiple-choice
   - Always (Value: 4)
   - Usually (Value: 3)
   - Sometimes (Value: 2)
   - Never (Value: 1)

3. **were you able to handle all the work you were assigned or expected to complete?**
   Key: *none*
   Format: Multiple-choice
   - Always (Value: 4)
   - Usually (Value: 3)
   - Sometimes (Value: 2)
   - Never (Value: 1)

Section 11. **Begin housework q9 - q10d**

1. **During the past 4 weeks (28 days), did you have any responsibility for housework or chores, such as cleaning, laundry, cooking, shopping, or caring for others, where you live?**
   Key: ANY_HOUSWORK_Q9
   Format: Multiple-choice
   - Yes (Value: 1)
   - No (Value: 2)

Section 12. **q9a**

Show if: (#ANY_HOUSWORK_Q9 IS '2')

1. **Which of the following was the main reason you did not have responsibility for housework or chores in the past 4 weeks (28 days)?**
   Key: *none*
   Format: Multiple-choice
   - Unable to do housework or chores because of my Multiple Sclerosis (Value: 1)
   - Away from home (Value: 2)
   - Others I live with are responsible for doing all housework or chores (Value: 3)

Section 13. **Q10 - NUM DAYS HOUSEWORK**

Show if: (#ANY_HOUSWORK_Q9 IS '1')

1. **During the past 4 weeks (28 days), how many days did you do any housework or chores?**
   Key: *none*
   Format: Free-form text

Please enter number of days here:

Section 14. **limit_housework_q10a**

Show if: (#ANY_HOUSWORK_Q9 IS '1')

1. **Did you limit the number of days you did housework or chores because of your Multiple Sclerosis?**
   Key: LIMIT_HOUSEWORK_10A
   Format: Multiple-choice
   - Yes (Value: 1)
   - No (Value: 2)

Section 15. **number days housework limited q10a followup**

Show if: (#LIMIT_HOUSEWORK_10A IS '1')

1. **How many additional days would you have done housework or chores?**
   Key: *none*
   Format: Free-form text

   Please enter days here:

Section 16. **limit hours of housework_q10b**

Show if: (#LIMIT_HOUSEWORK_10A IS-ANSWERED)

1. **On the days you did housework did you limit the number of hours you did housework or chores because of your Multiple Sclerosis?**
   Key: LIMIT_HOUSEWORK_Q10B
   Format: Multiple-choice
   - Yes (Value: 1)
   - No (Value: 2)

Section 17. **percent_housework_able_to_do_q10c**

Show if: (#LIMIT_HOUSEWORK_Q10B IS '1')

1. **About what percentage of the hours you needed to work to do housework or chores were you able to work during the past 4 weeks (28 days)?**
   Key: *none*
   Format: Free-form text

   Please enter the percent of needed hours you worked here:

Section 18. **hours_housework_q10d**

Show if: (#LIMIT_HOUSEWORK_Q10B IS-ANSWERED)

1. **On the days you did housework or chores, about how many hours did you work at housework or chores?**
   Key: *none*
   Format: Free-form text

   Please enter hours per day you did housework here:

Section 19. **housework_able_q10e**
On the days you did housework or chores: (Please check one answer for each)

Show if: (#LIMIT_HOUSEWORK_Q10B IS-ANSWERED)

1. **were you able to do your housework without making mistakes?**
   Key: *none*
   Format: Multiple-choice
   - Always (Value: 4)
   - Usually (Value: 3)
   - Sometimes (Value: 2)
   - Never (Value: 1)

2. **were you able to complete your housework on time?**
   Key: *none*
   Format: Multiple-choice
   - Always (Value: 4)
   - Usually (Value: 3)
   - Sometimes (Value: 2)
   - Never (Value: 1)

3. **were you able to handle all the housework you were expected to complete?**
   Key: *none*
   Format: Multiple-choice
   - Always (Value: 4)
   - Usually (Value: 3)
   - Sometimes (Value: 2)
   - Never (Value: 1)

Section 20. **MSRS_Q11**
For each of the following items, please select the level of disability that best represents your current condition.

1. **Walking**
   Key: *none*
   Format: Multiple-choice
   - No symptoms or disability in this specific area (Value: 1)
   - None - Aware of symptoms but no functional disability (Value: 2)
   - Mild - Mild disability but not requiring help from others (Value: 3)
   - Moderate - Moderate disability that requires some help from others (Value: 4)
   - Total - Total disability and help always required (Value: 5)

2. **Upper Limb Function**
   Key: *none*
   Format: Multiple-choice
   - No symptoms or disability in this specific area (Value: 1)
   - None - Aware of symptoms but no functional disability (Value: 2)
   - Mild - Mild disability but not requiring help from others (Value: 3)
   - Moderate - Moderate disability that requires some help from others (Value: 4)
   - Total - Total disability and help always required (Value: 5)

3. **Vision**
   Key: *none*
   Format: Multiple-choice
   - No symptoms or disability in this specific area (Value: 1)
   - None - Aware of symptoms but no functional disability (Value: 2)
   - Mild - Mild disability but not requiring help from others (Value: 3)
   - Moderate - Moderate disability that requires some help from others (Value: 4)
   - Total - Total disability and help always required (Value: 5)

4. **Speech**
   Key: *none*
   Format: Multiple-choice
   - No symptoms or disability in this specific area (Value: 1)
   - None - Aware of symptoms but no functional disability (Value: 2)
   - Mild - Mild disability but not requiring help from others (Value: 3)
   - Moderate - Moderate disability that requires some help from others (Value: 4)
   - Total - Total disability and help always required (Value: 5)

5. **Swallowing**
   Key: *none*
   Format: Multiple-choice
   - No symptoms or disability in this specific area (Value: 1)
   - None - Aware of symptoms but no functional disability (Value: 2)
   - Mild - Mild disability but not requiring help from others (Value: 3)
   - Moderate - Moderate disability that requires some help from others (Value: 4)
   - Total - Total disability and help always required (Value: 5)

6. **Thinking / Memory / Cognition**
   Key: *none*
   Format: Multiple-choice
   - No symptoms or disability in this specific area (Value: 1)
   - None - Aware of symptoms but no functional disability (Value: 2)
   - Mild - Mild disability but not requiring help from others (Value: 3)
   - Moderate - Moderate disability that requires some help from others (Value: 4)
   - Total - Total disability and help always required (Value: 5)

7. **Sensation / Burning / Pain**
   Key: *none*
   Format: Multiple-choice
   - No symptoms or disability in this specific area (Value: 1)
   - None - Aware of symptoms but no functional disability (Value: 2)
   - Mild - Mild disability but not requiring help from others (Value: 3)
   - Moderate - Moderate disability that requires some help from others (Value: 4)
   - Total - Total disability and help always required (Value: 5)

Section 21. **Current_treatment_q12**

1. **Which of the following are you currently taking to treat Multiple Sclerosis (MS)?**
   Key: CURRENT_DMT_Q12
   Format: Multiple-choice

   Please check one answer.

   - Copaxone (Glatiramer Acetate) (Value: 1)
   - Avonex or Avonex pre-filled syringe (Interferon Beta 1a - intramuscular) (Value: 2)
   - Rebif (Interferon Beta 1a - subcutaneous) (Value: 3)
   - Betaseron or Betaferon (Interferon Beta 1b - subcutaneous) (Value: 4)
   - Tysabri (Natalizumab) (Value: 5)
   - Novantrone (Mitoxantrone) (Value: 6)
   - None of the above (Value: 7)

Section 22. **first_treatment_q13**

Show if: (#CURRENT_DMT_Q12 IS-NOT '7')

1. **Is this the first treatment you have taken for your Multiple Sclerosis?**
   Key: IS_FIRST_DMT_Q13
   Format: Multiple-choice
   - Yes (Value: 1)
   - No (Value: 2)

Section 23. **previous_dmt_q13a**

Show if: (#IS_FIRST_DMT_Q13 IS '2')

1. **Which of the following did you take before your current treatment for Multiple Sclerosis?**
   Key: *none*
   Format: Check all that apply

   Please check all that you previously took.

   - Copaxone (Glatiramer Acetate) (Value: 1)
   - Avonex or Avonex pre-filled syringe (Interferon Beta 1a - intramuscular) (Value: 2)
   - Rebif (Interferon Beta 1a - subcutaneous) (Value: 3)
   - Betaseron or Betaferon (Interferon Beta 1b - subcutaneous) (Value: 4)
   - Tysabri (Natalizumab) (Value: 5)
   - Novantrone (Mitoxantrone) (Value: 6)

2. **Is your current treatment:**
   Key: *none*
   Format: Multiple-choice

   (Please check one answer; if more than one previous treatment please answer about the treatment you took

most recently)

- more effective than your previous treatment (Value: 1)
- less effective than your previous treatment (Value: 2)
- about the same effectiveness as your previous treatment (Value: 3)

3. **Compared with your current treatment, which of the following describes your experience of side effects with your previous treatment? Did you have:**
   Key: *none*
   Format: Multiple-choice

   (Please check one answer; if more than one previous treatment please answer about the treatment you took most recently)

   - the same side effects (Value: 1)
   - different side effects (Value: 2)
   - no side effects (Value: 3)

4. **Compared with your current treatment, how often did you experience side effects with your previous treatment?**
   Key: *none*
   Format: Multiple-choice

   (Please check one answer; if more than one previous treatment please answer about the treatment you took most recently)

   - More often (Value: 1)
   - Less often (Value: 2)
   - About the same (Value: 3)

Section 24. **current_treat_time_q14**

Show if: (#IS_FIRST_DMT_Q13 IS-ANSWERED)

1. **About how many months have you been taking your current medication to treat Multiple Sclerosis?**
   Key: TREAT_TIME_Q14
   Format: Multiple-choice

   If you stopped using the medication temporarily, answer with the total months you have used the medication. (Please check one answer)

   - Less than 1 month (Value: 1)
   - 1 to 3 months (Value: 2)
   - 4 to 6 months (Value: 3)
   - 7 to 9 months (Value: 4)
   - 10 to 12 months (Value: 5)
   - 13 to 18 months (Value: 6)
   - 19 to 24 months (Value: 7)
   - 25 or more months (Value: 8)

Section 25. **q14_specify**

Show if: (#TREAT_TIME_Q14 IS '8')

1. **How many months have you taken your current medication?**
   Key: *none*
   Format: Free-form text

   Please enter the number of months here:

Section 26. **q15_number_days**

Show if: (#CURRENT_DMT_Q12 IS-NOT '7')

1. **On how many days during the last 4 weeks (28 days) were you supposed to take this medication?**
   Key: DAYS_TO_TAKE_MED_Q15

Format: Multiple-choice

Please select number from the list.

- 0 (Value: 0)
- 1 (Value: 1)
- 2 (Value: 2)
- 3 (Value: 3)
- 4 (Value: 4)
- 5 (Value: 5)
- 6 (Value: 6)
- 7 (Value: 7)
- 8 (Value: 8)
- 9 (Value: 9)
- 10 (Value: 10)
- 11 (Value: 11)
- 12 (Value: 12)
- 13 (Value: 13)
- 14 (Value: 14)
- 15 (Value: 15)
- 16 (Value: 16)
- 17 (Value: 17)
- 18 (Value: 18)
- 19 (Value: 19)
- 20 (Value: 20)
- 21 (Value: 21)
- 22 (Value: 22)
- 23 (Value: 23)
- 24 (Value: 24)
- 25 (Value: 25)
- 26 (Value: 26)
- 27 (Value: 27)
- 28 (Value: 28)

Section 27. **q16 - miss any**

Show if: (#CURRENT_DMT_Q12 IS-NOT '7') AND (#DAYS_TO_TAKE_MED_Q15 IS-NOT '0')

1. **Did you miss or forget to take any doses of this medication during the last 4 weeks (28 days)?**
   Key: MISS_MEDS_Q16
   Format: Multiple-choice
   - Yes (Value: 1)
   - No (Value: 2)

Section 28. **num_doses_missed_q16a**

Show if: (#MISS_MEDS_Q16 IS '1')

1. **How many doses did you miss or forget?**
   Key: *none*
   Format: Free-form text

   Please enter number of doses here:

Section 29. **factors_miss_q16b**
How important were the following factors in missing or forgetting to take a dose? (Please check one answer for each)

Show if: (#MISS_MEDS_Q16 IS '1')

1. **Memory problems**
   Key: *none*
   Format: Multiple-choice
   - Extremely important (Value: 4)
   - Moderately important (Value: 3)
   - A little important (Value: 2)

- Not important at all (Value: 1)

2. **Too busy**
   Key: *none*
   Format: Multiple-choice
   - Extremely important (Value: 4)
   - Moderately important (Value: 3)
   - A little important (Value: 2)
   - Not important at all (Value: 1)

3. **Side effects of the injection**
   Key: *none*
   Format: Multiple-choice
   - Extremely important (Value: 4)
   - Moderately important (Value: 3)
   - A little important (Value: 2)
   - Not important at all (Value: 1)

4. **Side effects of the medication**
   Key: *none*
   Format: Multiple-choice
   - Extremely important (Value: 4)
   - Moderately important (Value: 3)
   - A little important (Value: 2)
   - Not important at all (Value: 1)

5. **Fear of needles**
   Key: *none*
   Format: Multiple-choice
   - Extremely important (Value: 4)
   - Moderately important (Value: 3)
   - A little important (Value: 2)
   - Not important at all (Value: 1)

6. **Needing someone to help me take my medication**
   Key: *none*
   Format: Multiple-choice
   - Extremely important (Value: 4)
   - Moderately important (Value: 3)
   - A little important (Value: 2)
   - Not important at all (Value: 1)

7. **Ran out of medication or could not refill my prescription**
   Key: *none*
   Format: Multiple-choice
   - Extremely important (Value: 4)
   - Moderately important (Value: 3)
   - A little important (Value: 2)
   - Not important at all (Value: 1)

8. **I was away from home and not able to access my medication**
   Key: *none*
   Format: Multiple-choice
   - Extremely important (Value: 4)
   - Moderately important (Value: 3)
   - A little important (Value: 2)
   - Not important at all (Value: 1)

9. **Feeling anxious, depressed, or nervous about taking my medication**
   Key: *none*
   Format: Multiple-choice
   - Extremely important (Value: 4)
   - Moderately important (Value: 3)
   - A little important (Value: 2)

- Not important at all (Value: 1)

10. **Dissatisfaction with my medication**
Key: *none*
Format: Multiple-choice
    - Extremely important (Value: 4)
    - Moderately important (Value: 3)
    - A little important (Value: 2)
    - Not important at all (Value: 1)

11. **I did not want taking my medication to interfere with my activities or responsibilities**
Key: *none*
Format: Multiple-choice
    - Extremely important (Value: 4)
    - Moderately important (Value: 3)
    - A little important (Value: 2)
    - Not important at all (Value: 1)

12. **Tired of taking my medication**
Key: *none*
Format: Multiple-choice
    - Extremely important (Value: 4)
    - Moderately important (Value: 3)
    - A little important (Value: 2)
    - Not important at all (Value: 1)

13. **Did not feel like taking my medication**
Key: *none*
Format: Multiple-choice
    - Extremely important (Value: 4)
    - Moderately important (Value: 3)
    - A little important (Value: 2)
    - Not important at all (Value: 1)

Section 30. **grasp_injector_q17**

Show if: ((#CURRENT_DMT_Q12 IS '1') OR (#CURRENT_DMT_Q12 IS '2') OR (#CURRENT_DMT_Q12 IS '3') OR (#CURRENT_DMT_Q12 IS '4') OR (#CURRENT_DMT_Q12 IS '6')) AND (#DAYS_TO_TAKE_MED_Q15 IS-NOT '0')

1. **During the past 4 weeks (28 days) have you had any difficulties with your ability to grasp or hold your injector?**
Key: *none*
Format: Multiple-choice
    - None (Value: 1)
    - Mild (Value: 2)
    - Moderate (Value: 3)
    - Severe (Value: 4)

Section 31. **cope_q18**
During the past 4 weeks (28 days) did you usually... (Please check yes or no for each)

Show if: (#CURRENT_DMT_Q12 IS-NOT '7') AND (#DAYS_TO_TAKE_MED_Q15 IS-NOT '0')

1. **use ice, a cold pack or a could compress on the injection site prior to taking your treatment?**
Key: *none*
Format: Multiple-choice
    - Yes (Value: 1)
    - No (Value: 2)

2. **use heat, a heat pack, or a hot compress on the injection site prior to taking your treatment?**
Key: *none*
Format: Multiple-choice
    - Yes (Value: 1)
    - No (Value: 2)

3. **take oral medications for pain relief, such as Aleve, Ibuprofen, Tylenol, aspirin, prior to taking your treatment?**
   Key: *none*
   Format: Multiple-choice
   - Yes (Value: 1)
   - No (Value: 2)

4. **use a cream, ointment, or lotion at the injection site for pain relief when you took your treatment?**
   Key: *none*
   Format: Multiple-choice
   - Yes (Value: 1)
   - No (Value: 2)

5. **take an anti-histamine to help control rashes or swelling when you took your treatment?**
   Key: *none*
   Format: Multiple-choice
   - Yes (Value: 1)
   - No (Value: 2)

6. **use a cream, ointment, or lotion at the injection site for relief from itching when you took your treatment?**
   Key: *none*
   Format: Multiple-choice
   - Yes (Value: 1)
   - No (Value: 2)

7. **schedule administration of your treatment so that it would not interfere with work or similar responsibilities?**
   Key: *none*
   Format: Multiple-choice
   - Yes (Value: 1)
   - No (Value: 2)

8. **schedule administration of your treatment so that it would not interfere with your leisure time, or recreational or social activities?**
   Key: *none*
   Format: Multiple-choice
   - Yes (Value: 1)
   - No (Value: 2)

9. **schedule administration of your treatment so that side effects would not interfere with work or similar responsibilities?**
   Key: *none*
   Format: Multiple-choice
   - Yes (Value: 1)
   - No (Value: 2)

10. **schedule administration of your treatment so that side effects would not interfere with your leisure time, or recreational or social activities?**
   Key: *none*
   Format: Multiple-choice
   - Yes (Value: 1)
   - No (Value: 2)

11. **massage the injection site after your treatment to relieve pain, swelling, itching or other discomfort?**
   Key: *none*
   Format: Multiple-choice
   - Yes (Value: 1)
   - No (Value: 2)

12. **manage to take the entire dose of treatment as recommended**
   Key: *none*
   Format: Multiple-choice

- Yes (Value: 1)
- No (Value: 2)

Section 32. **experience_q19**
During the past 4 weeks (28 days) did you... (Please check one answer for each)

Show if: (#DAYS_TO_TAKE_MED_Q15 IS-NOT '0') AND (#CURRENT_DMT_Q12 IS-NOT '7')

1. **have bleeding at the injection site?**
   Key: *none*
   Format: Multiple-choice
   - Never (Value: 1)
   - A few times (Value: 2)
   - About half the time (Value: 3)
   - Most of the time (Value: 4)
   - All or nearly all the time (Value: 5)

2. **have pain, stinging, burning, or soreness at the injection site during administration of your treatment?**
   Key: *none*
   Format: Multiple-choice
   - Never (Value: 1)
   - A few times (Value: 2)
   - About half the time (Value: 3)
   - Most of the time (Value: 4)
   - All or nearly all the time (Value: 5)

3. **have itching or irritation at the injection site during administration of your treatment?**
   Key: *none*
   Format: Multiple-choice
   - Never (Value: 1)
   - A few times (Value: 2)
   - About half the time (Value: 3)
   - Most of the time (Value: 4)
   - All or nearly all the time (Value: 5)

4. **feel nervous or anxious before or during administration of your treatment?**
   Key: *none*
   Format: Multiple-choice
   - Never (Value: 1)
   - A few times (Value: 2)
   - About half the time (Value: 3)
   - Most of the time (Value: 4)
   - All or nearly all the time (Value: 5)

5. **have pain, burning, stinging, or soreness at the injection site after administration of your treatment?**
   Key: *none*
   Format: Multiple-choice
   - Never (Value: 1)
   - A few times (Value: 2)
   - About half the time (Value: 3)
   - Most of the time (Value: 4)
   - All or nearly all the time (Value: 5)

6. **have irritation or itching at the injection site after administration of your treatment?**
   Key: *none*
   Format: Multiple-choice
   - Never (Value: 1)
   - A few times (Value: 2)
   - About half the time (Value: 3)
   - Most of the time (Value: 4)
   - All or nearly all the time (Value: 5)

7. **have swelling, welts, or lumps at the injection site after administration of your treatment?**

none
Format: Multiple-choice

- Never (Value: 1)
- A few times (Value: 2)
- About half the time (Value: 3)
- Most of the time (Value: 4)
- All or nearly all the time (Value: 5)

8. **have abnormal redness of the skin or a rash at the injection site after administration of your treatment?**
   Key: *none*
   Format: Multiple-choice
   - Never (Value: 1)
   - A few times (Value: 2)
   - About half the time (Value: 3)
   - Most of the time (Value: 4)
   - All or nearly all the time (Value: 5)

9. **have bruises at the injection site after administration of your treatment?**
   Key: *none*
   Format: Multiple-choice
   - Never (Value: 1)
   - A few times (Value: 2)
   - About half the time (Value: 3)
   - Most of the time (Value: 4)
   - All or nearly all the time (Value: 5)

10. **have chills, headaches, or flu-like symptoms after your treatment?**
    Key: *none*
    Format: Multiple-choice
    - Never (Value: 1)
    - A few times (Value: 2)
    - About half the time (Value: 3)
    - Most of the time (Value: 4)
    - All or nearly all the time (Value: 5)

11. **have confidence that your treatment was properly administered?**
    Key: *none*
    Format: Multiple-choice
    - Never (Value: 1)
    - A few times (Value: 2)
    - About half the time (Value: 3)
    - Most of the time (Value: 4)
    - All or nearly all the time (Value: 5)

Section 33. **skin_probs_q20**

Show if: (#CURRENT_DMT_Q12 IS-NOT '7')

1. **During the past 4 weeks (28 days) have you experienced any lumps, bumps, or dents in your skin or loss of skin at any injection sites?**
   Key: *none*
   Format: Multiple-choice

   (Please check one answer)

   - Yes (Value: 1)
   - No (Value: 2)

Section 34. **expect_q21_q22**

Show if: (#DAYS_TO_TAKE_MED_Q15 IS-NOT '0') AND (#CURRENT_DMT_Q12 IS-NOT '7')

1. **Considering any problems or discomfort you had DURING the administration of your treatment during the past 4 weeks (28 days), were these more of a problem, less of a problem, or about**

what you expected?
Key: *none*
Format: Multiple-choice

- More of a problem (Value: 1)
- Less of a problem (Value: 2)
- About what I expected (Value: 3)

2. **Considering any problems or discomfort you had AFTER the administration of your treatment during the past 4 weeks (28 days), were these more of a problem, less of a problem, or about what you expected?**
   Key: *none*
   Format: Multiple-choice
   - More of a problem (Value: 1)
   - Less of a problem (Value: 2)
   - About what I expected (Value: 3)

Section 35. **inject_q23_q24**

Show if: ((#CURRENT_DMT_Q12 IS '1') OR (#CURRENT_DMT_Q12 IS '2') OR (#CURRENT_DMT_Q12 IS '3') OR (#CURRENT_DMT_Q12 IS '4') OR (#CURRENT_DMT_Q12 IS '6')) AND (#DAYS_TO_TAKE_MED_Q15 IS-NOT '0')

1. **During the past 4 weeks (28 days) did you manually inject, use an auto-injection device, or do both?**
   Key: *none*
   Format: Multiple-choice
   - Manual injection only (Value: 1)
   - Auto injection only (Value: 2)
   - Both (Value: 3)

2. **During the past 4 weeks (28 days), how often was your injection done by someone else?**
   Key: *none*
   Format: Multiple-choice
   - Never (Value: 1)
   - A few times (Value: 2)
   - About half the time (Value: 3)
   - Most of the time (Value: 4)
   - All or nearly all the time (Value: 5)

Section 36. **support_serv_q25**

Show if: (#CURRENT_DMT_Q12 IS-NOT '7')

1. **During the past 4 weeks (28 days), how many times have you called the manufacturer's patient support services for the treatment you were taking?**
   Key: *none*
   Format: Multiple-choice
   - None - I didn't know about the service (Value: 1)
   - None - I didn't need the service (Value: 2)
   - Once or twice (Value: 3)
   - 2 or 3 times (Value: 4)
   - 4 or more times (Value: 5)

Section 37. **avoid_sites_q26**
During the past 4 weeks (28 days), have you avoided use of any injection sites for any of the following reasons?

Show if: ((#CURRENT_DMT_Q12 IS '1') OR (#CURRENT_DMT_Q12 IS '2') OR (#CURRENT_DMT_Q12 IS '3') OR (#CURRENT_DMT_Q12 IS '4') OR (#CURRENT_DMT_Q12 IS '6')) AND (#DAYS_TO_TAKE_MED_Q15 IS-NOT '0')

1. **Irritation or skin problems at the site**
   Key: *none*
   Format: Multiple-choice
   - Yes (Value: 1)
   - No (Value: 2)

2. **Difficult to reach**
   Key:

none
Format: Multiple-choice

- Yes (Value: 1)
- No (Value: 2)

3. **Pain**
   Key: *none*
   Format: Multiple-choice
   - Yes (Value: 1)
   - No (Value: 2)

4. **Doesn’t work well with current needle length or depth of injection**
   Key: *none*
   Format: Multiple-choice
   - Yes (Value: 1)
   - No (Value: 2)

5. **Doesn’t work well with current needle gauge or thickness**
   Key: *none*
   Format: Multiple-choice
   - Yes (Value: 1)
   - No (Value: 2)

6. **Doesn’t work well with speed of injection**
   Key: *none*
   Format: Multiple-choice
   - Yes (Value: 1)
   - No (Value: 2)

Section 38. **last_q27_q30**

Show if: (#CURRENT_DMT_Q12 IS-NOT '7')

1. **Which of the following best describes what you expected when you first started taking your current treatment?**
   Key: *none*
   Format: Multiple-choice

   (Please check one answer)

   - It would prevent me from having any relapses and make me feel better (Value: 1)
   - It would prevent me from having any relapses without making me feel better (Value: 2)
   - It would reduce the frequency of my relapses by about 50% or more (Value: 3)
   - It would reduce the frequency of my relapses by about 50% or less (Value: 4)
   - I did not think the treatment would have any effect on my relapses (Value: 5)

2. **Which of the following best describes the effectiveness of your current treatment?**
   Key: *none*
   Format: Multiple-choice

   (Please check one answer)

   - It has prevented me from having any relapses and makes me feel better (Value: 1)
   - It has prevented me from having any relapses but it does not make me feel better (Value: 2)
   - It has reduced the frequency of my relapses by about 50% or more (Value: 3)
   - It has reduced the frequency of my relapses by about 50% or less (Value: 4)
   - I do not think the treatment has had any effect on my relapses (Value: 5)

3. **Overall, how hard or easy do you feel it is to take your current Multiple Sclerosis treatment as recommended by your physician?**
   Key: *none*
   Format: Multiple-choice

   (Please check one answer)

   - Extremely easy (Value: 1)

- A little hard (Value: 2)
- Moderately hard (Value: 3)
- Very hard (Value: 4)
- Extremely hard (Value: 5)

4. **Overall, how satisfied are you with how things have been with your treatment during the past 4 weeks (28 days)?**
   Key: *none*
   Format: Multiple-choice

   (Please check one answer)

   - Not satisfied at all (Value: 1)
   - A little satisfied (Value: 2)
   - Moderately satisfied (Value: 3)
   - Very satisfied (Value: 4)
   - Completely satisfied (Value: 5)

Section 39. **open_end**

Show if: (#CURRENT_DMT_Q12 IS-ANSWERED)

1. **Thank you. Those are all the questions we have. If there is anything else you think is important for us to know, or that will help us understand your responses, please tell us in the space below.**
   Key: *none*
   Format: Free-form text

   When you are finished, please 'submit' your survey.

Generated Dec 30, 2009 01:58PM

© 2005-2009 PatientsLikeMe. Information on PatientsLikeMe.com does not constitute medical advice.
