# Supplementary material for: Use of an Online Community to Develop Patient-Reported Outcome Instruments: The Multiple Sclerosis Treatment Adherence Questionnaire (MS-TAQ)
Source: J Med Internet Res. 2011 Jan 24;13(1):e12. doi: 10.2196/jmir.1687 (PMC3221333; doi:10.2196/jmir.1687)
Supplement: Supplementary file 2 [file jmir_v13i1e12_app2.pdf]

# Multiple Sclerosis Treatment Experience Questionnaire (MSTEQ)

Name:__________________________________________________

Today's date (mm/dd/yy):________________________________

It is important that you take your medication as prescribed by your physician. However, from time to time you may find it difficult or impossible to take your DMT as prescribed. This instrument is designed to help you and your physician understand what these barriers are and to come up with ways to make it easier to take your treatment. It is very important that you are honest when completing this instrument so that your MS team can help to look after you better.

1. ) Which of the following are you currently taking to treat Multiple Sclerosis (MS)? (Check one)

| | |
|---|---|
| Copaxone (Glatiramer Acetate) | |
| Avonex or Avonex pre-filled syringe (Interferon Beta 1a - intramuscular) | |
| Rebif (Interferon Beta 1a - subcutaneous) | |
| Betaseron or Betaferon (Interferon Beta 1b - subcutaneous) | |
| Tysabri (Natalizumab) | |
| Novantrone (Mitoxantrone) | |
| Other, please specify: | |

2.) On how many days during the last 4 weeks (28 days) were you supposed to take this medication? (Check one)

| | |
|---|---|
| Every day (28 times) | |
| Every other day (14 times) | |
| Three times a week (12 times) | |
| Once a week (4 times) | |
| Once a month (1 time) | |
| Other, please specify: | |

2.) During the past 4 weeks (28 days) did you manually inject, use an auto-injection device, or do both? (Check one)

| | |
|---|---|
| Manual injection only | |
| Auto injection only | |
| Both manual and auto-injection | |
| Not applicable / I take a pill | |

3.) During the past 4 weeks (28 days), how often was your injection done by someone else? (Check one)

| | |
|---|---|
| Never | |
| A few times | |
| About half the time | |
| Most of the time | |
| All or nearly all of the time | |

4.) Did you miss or forget to take any doses of this medication during the last 4 weeks (28 days)? (Check one)

| | |
|---|---|
| Yes | |
| No | |

5.) How many doses did you miss or forget? (Complete blank)

---

**IF YOU HAVEN'T SKIPPED ANY DOSES IN THE PAST 28 DAYS, SKIP TO QUESTION 7**

**(Complete this section only if you missed a dose in the past 28 days)**

6. How important were the following factors in missing or forgetting to take a dose? (Please check one answer for each)

| | Not important at all | A little important | Moderately important | Extremely important |
|---|---|---|---|---|
| Memory problems | 0 | 1 | 2 | 3 |
| Too busy | 0 | 1 | 2 | 3 |
| Side effects of injection | 0 | 1 | 2 | 3 |
| Side effects of medication | 0 | 1 | 2 | 3 |
| Fear of needles | 0 | 1 | 2 | 3 |
| Needing someone to help me take my medication | 0 | 1 | 2 | 3 |
| Ran out of medication or could not refill my prescription | 0 | 1 | 2 | 3 |
| Away from home and could not access my medication | 0 | 1 | 2 | 3 |
| Feeling anxious, depressed, or nervous about taking my medication | 0 | 1 | 2 | 3 |
| Dissatisfaction with my medication | 0 | 1 | 2 | 3 |
| Did not want taking my medication to interfere with activities | 0 | 1 | 2 | 3 |
| Tired of taking my medication | 0 | 1 | 2 | 3 |
| Did not feel like taking my medication | 0 | 1 | 2 | 3 |

For completion by examiner only; DMT-Barr Score:___________

7. During the past 4 weeks (28 days) did you… (Please check one answer for each)

| | Never | A few times | About half the time | Most of the time | All or nearly all the time |
|---|---|---|---|---|---|
| Have bleeding at the injection site? | 0 | 1 | 2 | 3 | 4 |
| Have pain, stinging, burning, or soreness at the injection site during administration of your treatment? | 0 | 1 | 2 | 3 | 4 |
| Have itching or irritation at the injection site during administration of your treatment? | 0 | 1 | 2 | 3 | 4 |
| Feel nervous or anxious during administration of your treatment? | 0 | 1 | 2 | 3 | 4 |
| Have pain, stinging, burning, or soreness at the injection site after administration of your treatment? | 0 | 1 | 2 | 3 | 4 |
| Have itching or irritation at the injection site after administration of your treatment? | 0 | 1 | 2 | 3 | 4 |
| Have swelling, welts, or lumps at the injection site after administration of your treatment? | 0 | 1 | 2 | 3 | 4 |
| Have abnormal redness of the skin or a rash at the injection site after administration of your treatment? | 0 | 1 | 2 | 3 | 4 |
| Have bruises at the injection site after administration of your treatment? | 0 | 1 | 2 | 3 | 4 |
| Have chills, headaches, or flu-like symptoms after your treatment? | 0 | 1 | 2 | 3 | 4 |

For completion by examiner only; DMT-SE Score:___________

8. During the past 4 weeks (28 days) did you usually… (Please check one answer for each)

| | No | Yes |
|---|---|---|
| Use ice, a cold pack or a cold compress on the injection site prior to taking your treatment? | 0 | 1 |
| Use heat, a heat pack, or a hot compress on the injection site prior to taking your treatment? | 0 | 1 |
| Take oral medications for pain relief, such as Aleve, Ibuprofen, Tylenol, aspirin, prior to taking your treatment? | 0 | 1 |
| Use a cream, ointment, or lotion at the injection site for pain relief when you took your treatment? | 0 | 1 |
| Take an anti-histamine to help control rashes or swelling when you took your treatment? | 0 | 1 |
| Use a cream, ointment, or lotion at the injection site for relief from itching when you took your treatment? | 0 | 1 |
| Massage the injection site after your treatment to relieve swelling, itching or other discomfort? | 0 | 1 |

For completion by examiner only; DMT-COPE Score:___________

9.) Overall, how hard or easy do you feel it is to take your current Multiple Sclerosis treatment as recommended by your physician? (Check one)

| | |
|---|---|
| Extremely easy | 1 |
| A little hard | 2 |
| Moderately hard | 3 |
| Very hard | 4 |
| Extremely hard | 5 |

10.) Overall, how satisfied are you with how things have been with your treatment during the past 4 weeks (28 days)? (Check one)

| | |
|---|---|
| Not satisfied at all | 1 |
| A little satisfied | 2 |
| Moderately satisfied | 3 |
| Very satisfied | 4 |
| Completely satisfied | 5 |

Thank you for completing this survey, you are now finished. Please check over your answers carefully, then return the completed form to the examiner.
